# Legislating for transnational ageing: a challenge to the logics of the welfare state

**DOI:** 10.1007/s10433-017-0431-6

**Published:** 2017-05-17

**Authors:** Anita Böcker, Alistair Hunter

**Affiliations:** 1 Centre for Migration Law, Radboud University, PO Box 9049, 6500 KK Nijmegen, The Netherlands; 2 The Alwaleed Centre, University of Edinburgh, 16 George Square, Edinburgh, EH8 9LD UK

**Keywords:** Transnational ageing, Return migration aids, Older migrants, Territoriality, Nationality, France, The Netherlands

## Abstract

Transnational ageing presents fundamental challenges to nationally bounded welfare states, which historically have tended to be organised according to a logic of solidarity among nationals and permanent residents of a given state territory. Nonetheless, the Dutch and French governments have taken steps to break this link between solidarity and territorially bounded consumption of welfare, by providing lifelong income security for older migrants who return to countries of origin on a permanent or semi-permanent basis. This article asks what motivated policymakers to initially develop these novel policy tools for transnational ageing which contradict the territorial logic of the welfare state. Based on interviews with key stakeholders and available official documents, we find that in both France and the Netherlands, policymakers’ initial motivations can be characterised as rather benign, if not beneficent: to facilitate return for those who are willing but unable to afford it. However, two types of obstacle have impeded the delivery of such policies. Non-discrimination clauses and free movement rights in EU law may make it difficult to implement policies for specific categories of older migrants. Electoral *realpolitik* may also lead policymakers to shelve policies which benefit older migrants, in a European context where public opinion on immigration is less and less favourable. Nonetheless, opposition may be neutralised by the budgetary advantages of these schemes, since older returnees do not consume public services such as healthcare.

## Introduction

Transnational ageing has self-evident implications for social protection^1^ insofar as national borders function as institutionalised ‘thresholds of inequality’ between different welfare regimes (Stichweh 1998, cited in Bommes 2000: p. 91). The term transnationalism seeks to capture ‘the *frequent* and *durable* participation of immigrants in the economic, political, and cultural life of their countries [of residence and origin], which requires regular and frequent contact across national borders’ (Portes et al. 2007; emphasis added). While there is now ample evidence that many older migrants engage in transnational activities and relationships, it is important to acknowledge that not *all* older migrants do so. Furthermore, ageing transnationally may be situational or temporary, take different forms, and—importantly—does not always imply physical mobility across borders, as seen for example when the care arrangements for ‘left-behind’ ageing parents are coordinated from abroad by their émigré children (Baldassar et al. 2007). However, the literature on transnational ageing has mostly focused on older people who physically migrate across borders as part of their transnational repertoires, and retirement constitutes a favourable moment for migration, due to the ending of the sedentary constraint of participation in the labour market. This literature has distinguished three main forms of late-in-life migration (Warnes et al. 2004), and for each we find numerous examples of regular and durable cross-border ties.

‘Amenity-seeking’ migrants are relatively affluent older individuals who relocate on the basis of factors such as climate, scenery or ‘lifestyle’. Studies have documented how amenity migrants develop transnational lifestyles and identities, often dividing their time between their retirement country and their ‘home country’ and claiming to feel ‘at home’ in both places (Gustafson 2008). ‘Family-joining’ migrants, also referred to as the ‘zero-generation’, may temporarily move to be closer to adult children who emigrated previously. These seniors often have a key role in transmitting the home country language and culture to their grandchildren (Nedelcu 2009). ‘Retirement return’ migrants, the focus of this paper, are former labour migrants who move on a permanent or temporary (dual residence) basis back to places of origin at retirement. Among those who return definitively, their ageing may be transnational insofar as they receive their state pension income from—and often remain attached in other ways as well with—their former country of residence, as the case presented here of Turks and Moroccans returning from the Netherlands demonstrates (Balkir and Böcker 2015; De Bree et al. 2010). However, definitive return appears to be the exception rather than the rule, as representative survey data from France and Switzerland show: more common is the dual residence strategy (Attias-Donfut 2005; Bolzman et al. 2006). Our second case study focuses on migrants whose ageing is archetypally transnational, namely migrant worker hostel residents circulating between France and places of origin in North and West Africa.

**Fig. 1:**
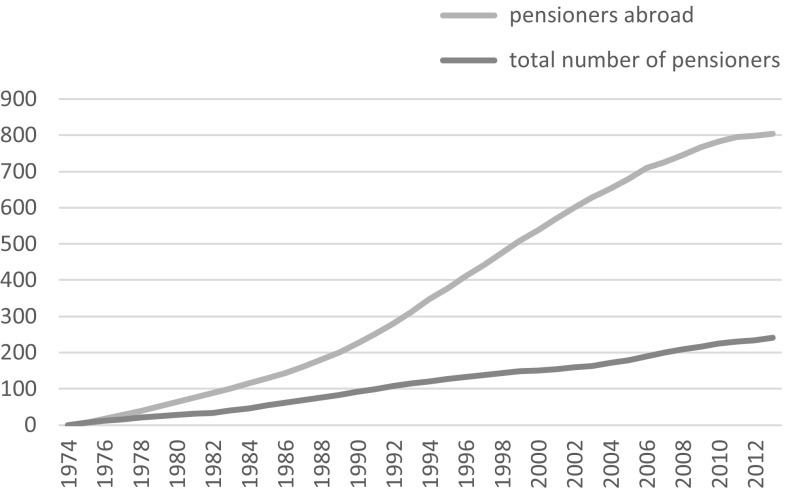
French pensions: growth in number of recipients (% change relative to 1974) *Source* Caisse nationale de l’assurance vieillesse 2013

**Fig. 2:**
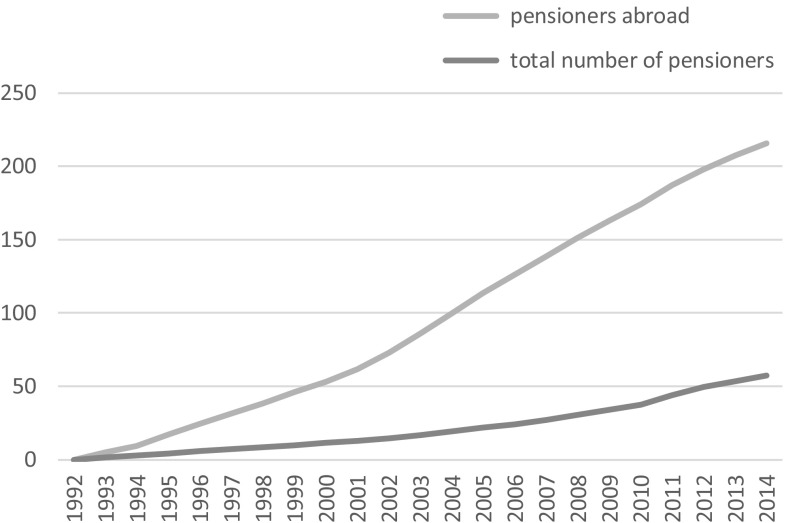
Dutch pensions: growth in number of recipients (% change relative to 1992) *Source* Sociale Verzekeringsbank

The exportability of state pensions thanks to bilateral social security accords may facilitate the transnational ageing of those whose transnationalism includes physical cross-border mobility, and statistics compiled by many OECD countries show the growing number of pensions paid to beneficiaries who reside abroad (see Figs. 1, 2). However, other forms of social protection are much less exportable, implying significant constraints to transnational ageing. These constraints arise due to the territorial and nationalistic logics of the welfare state, which we will unpack in the next section. In this paper, we focus on two pieces of legislation which invert these welfare state principles by providing lifelong income security to non-nationals following their return to countries of origin. In the Netherlands, such a scheme has been in existence since 1985. In France, analogous legislation was voted by parliament in 2007, eventually coming into force in January 2016. The puzzle this paper addresses is what motivated policy makers to draft and implement such legislation during a period when the welfare state has in most other respects been in retreat. We subsequently chart the development of the two pieces of legislation over time. We conclude prospectively by evaluating these two policy measures to facilitate transnational ageing and examine the potential for such tools to be adopted by other countries where significant numbers of older migrants live.

## The logics of the welfare state: solidarity, territoriality, nationality

Welfare states have been characterised as inherently closed systems by both migration and social security scholars (e.g. Freeman 1986; Myrdal 1960; Pennings 2015; Sciortino and Finotelli 2015). De Swaan (1988) described the evolution of the modern welfare state in Europe as a process of collectivisation of care that went hand in hand with the process of state formation. Collective arrangements for poor relief and other social provisions were initially brought about at the parish level, in early modernity at the regional level, and in the late modern era at the level of nation-states. However, De Swaan was rather pessimistic about the prospects for transnational social policy: ‘[A] welfare state is not only a national system, but it is also anti-international: a socially secure society is also a closed society’ (De Swaan 1995: p. 9). In a recent study, Faist and Bilecen doubted whether social inequalities in Europe are bound to translate into a transnational social question: ‘For the fact is that over the past four decades, the opening of national borders for the common market in the EU in the sense of ‘negative integration’ has not been rectified by “positive integration” measures and the creation of more uniform social standards’ (Faist and Bilecen 2015: p. 291).

Welfare states and their social protection systems are usually based on three principles: solidarity, territoriality and nationality. Van der Mei (2003) defined the welfare state as a political community whose members have decided to set a number of social objectives which the market is unable to achieve. In order to achieve these objectives, the market principle of distribution according to individual economic performance must be partly replaced by forms of redistribution that require generalised reciprocity or diffuse solidarity. This presumes, however, ‘the existence of boundaries that distinguish those who are members of the community from those who are not’ (Freeman 1986: p. 52). This is where the two other principles come in, and why the transnational ageing of retired labour migrants represents challenges to welfare states.

The principle of territoriality implies, first, that welfare state benefits are preserved for persons residing or working on the state territory, and secondly, that they must be consumed on the state territory (Halfmann 2000; Van der Mei 2003). For cash benefits, territoriality implies that they cannot be transferred abroad. In practice, old-age pensions—financed by an individual’s contributions—are often exportable, whereas many other benefits (e.g. unemployment benefits) and particularly non-contributory benefits (which are financed by general taxation) are not. Even within the European Union, social assistance benefits and so-called special non-contributory benefits are provided exclusively in the member state in which the beneficiary resides and are therefore not exportable.

One reason for the non-portability of benefits is that outmigration to another state is interpreted as giving up membership and hence entitlement to benefits (Van der Mei 2003). Welfare states, to paraphrase Bommes (2000), provide an ordered lifecourse by protecting against life events such as unemployment, ill-health or retirement. This is based on assumptions of ‘a more or less static and bounded populace and a ‘normal’ lifecourse that proceeds from a phase of contributions to a phase of claims’ (Ackers and Dwyer 2004: p. 463). Migrant lifecourses, however, are likely to diverge from these assumptions. For example, migrants are often not able to build up full pension rights, because pension laws require that a person has lived or worked on the state territory for, e.g. 40 years, or—as is the case in the Netherlands—between the ages of 15 and 65, to be entitled to a full pension in his or her old age. Among the foreign-born pensioners in the Netherlands in 2015, only 27% were receiving a full state pension, as compared to 95% of the native-born pensioners.^2^ Since low-skilled migrants often work in poorly paid sectors in which they cannot accumulate sufficient occupational pensions or private savings, many are dependent on non-contributory income support benefits—which cannot be transferred abroad. For example, in the Netherlands, recipients of income support are allowed to stay abroad for only 13 weeks a year. In France, where the proportion of foreign-born recipients of old-age income support is nearly 40% (while foreign-born make up only 8% of the total population aged over 65), one is required to spend a minimum of 6 months per year on French territory. In effect, welfare states ‘timetable’ the lives of older people who are dependent on social protection (Hunter 2016).

Welfare states evolved within the legal-political framework of nation-states, and nation-states have a specific bond with their own nationals. They therefore tended to make entitlement to non-contributory benefits conditional not only on residence or employment in the state territory but also upon the nationality of the state. In the nineteenth century, generally it was the sending state, not the host state, which was held responsible for supporting needy migrants (Vonk 2002). Since the second half of the twentieth century, nationality requirements have increasingly been replaced by residence requirements. Domestic as well as international legal norms have sharply reduced welfare states’ room for excluding long-term legal residents (the so-called denizens) from social protection (Sciortino and Finotelli 2015: p. 187). However, migrants without permanent residence status may still lose their residence rights if they apply for (non-contributory) income support benefits (Vonk 2002).

The distinction between nationals and non-nationals is prominently reflected in national immigration laws and policies. International law obliges states to admit their own nationals to their territory—and nationals who actually establish residence there are usually offered access to the state’s welfare system, but states are in principle free to deny and regulate the access of non-nationals to their territory. In determining whether and on what conditions non-nationals will be admitted, welfare states consider whether prospective immigrants will be net contributors or net beneficiaries (cf. Sciortino and Finotelli 2015). This implies, for example, that economically inactive persons are required to have sufficient means to provide for themselves. Also within the European Union, economically inactive EU citizens must have ‘sufficient resources not to become a burden on the social assistance system of the host member state’ during the first 5 years of residence and recent case law of the European Court of Justice shows a tendency towards a restrictive interpretation of this requirement. Commentators have argued that this makes it effectively impossible for pensioners from poorer member states to move to richer member states and enjoy the other rights that come with EU citizenship (Meduna et al. 2014; Mantu and Minderhoud 2016).

In sum, welfare states are territorially operating systems based on a form of membership which is defined in terms of nationality and/or residence on the territory. Our research question focuses on two policies in France and the Netherlands which invert these principles. The next section gives some background to the French and Dutch cases and details the methods we deployed.

## Research context and methods

The Dutch remigration scheme has been in operation since 1985. It guarantees a basic monthly income to older migrants who wish to resettle in their country of origin. Since its introduction, on average 400–500 migrant households have left the Netherlands with a remigration benefit each year, with Turkey and Morocco being the most important destinations. Beneficiaries receive a monthly payment which tops up the income of Turkish and Moroccan couples to €525 and €600, respectively. In 2015, approximately 7000 households received a remigration benefit. Another 7000 households had dormant rights.^3^ The French scheme, the Aid for Familial and Social Reinsertion, entered into force in January 2016. Beneficiaries receive a yearly payment which tops up their annual pension income to €6,600 (equivalent to €550 per month). Given its recent introduction, no statistics are yet available regarding the number of beneficiaries. However, in interviews migrant welfare NGOs indicated that the take-up of the aid thus far has been minimal. The Ministry of Social Affairs, responsible for drafting the law, estimated that some 35,000 people are eligible for the Aid.

These two schemes are part of a larger family of ‘return aids’. During and after the economic recession of the 1970s, various Western European countries implemented schemes to encourage ‘guest workers’ from the Mediterranean region to return home (Petek-Salom 2002). The incentives offered could include in-kind assistance such as business start-up advice or vocational training, as well as cash payments such as ‘return bonuses’ or refunds of pension and unemployment insurance contributions that had been paid by migrants during their stay in the host country (IOM 2004). Since the 1990s, many European countries have established ‘pay-to-go schemes’ that target migrants without legal residence, in particular failed asylum seekers (Black et al. 2011; OECD 2008). In the wake of the 2008 recession, Spain implemented a scheme which offers unemployed migrants from twenty countries who wish to return home the possibility to receive all their unclaimed unemployment benefits (Pabón López and Davis 2011). Though the target groups, policy goals and other specifics vary, these schemes generally offer financial incentives in the form of one-off lump sums. The two schemes we focus on here are different, however, insofar as they provide lifelong income security following return. As far as we have been able to determine, only Denmark has a similar scheme, which targets older refugees and labour migrants who wish to return to their home country but are unable to support themselves (ECRE 2005; OECD 2011).

In terms of research design, we chose to focus on the French and Dutch schemes. Our central research question asks what motivated French and Dutch policymakers to develop policies for transnational ageing which reverse the narrowly territorial and nationalistic logics of the welfare state, particularly during a period of welfare state retrenchment. The data we analyse in order to answer this question are of two types. Primarily, we have relied on an exhaustive documentary analysis of the publicly available documentation regarding the two return aids. This includes legislative texts, parliamentary or government reports, transcripts of parliamentary debates and sub-committees, ministerial press releases, information from the websites of agencies charged with implementing the legislation, as well as news coverage. This documentary evidence is supplemented by 10 semi-structured qualitative interviews with stakeholders selected because of their familiarity with the legislation, such as members of parliament, government officials and representatives of migrant rights organisations. The interview data cited in the paper primarily refer to the French case, as this legislation is very new and there is a lack of publicly available information about it, in contrast with the Dutch policy. Some interviewees were recorded, while others preferred to speak off the record, in which case extensive notes were taken at the time of the interview. For both types of source, the aim of our analysis was to uncover patterns in the motivations of policymakers legislating in this area over time, as will be presented in the next section.

## Results

Table 1 summarises the development over time of the Dutch Remigration Benefit and the French Aid for Familial and Social Reinsertion. From this overview, a certain number of similarities and differences between the two schemes become apparent. As regards similarities, both aids are restricted to non-nationals who have modest incomes and who are retired or who are approaching retirement age (although the lower age limit for the Dutch aid has varied in this regard). There are also several important differences between the schemes. The first to note is that the Dutch scheme requires permanent return, whereas the French scheme facilitates extended return visits of at least 6-month duration per year. Secondly, the residence criteria for the Dutch scheme are more inclusive than those for the French scheme. The latter very selectively targets long-term residents living in accommodation with a specific legal status (*foyers de travailleurs migrants* and *résidences sociales*). In what follows, we analyse the genesis and subsequent development of each scheme. We conclude by assessing the prospects elsewhere for future policy measures designed to promote transnational ageing.

**Table 1:** Characteristics of the Dutch remigration scheme and the French aid for familial and social reinsertion scheme

	NL-Pilot remigration scheme (1985)	NL-Remigration Act (1999)	NL-Revised Remigration Act (2014)	FR-ARFS (2007) Act of Parliament	FR-ARFS (2016) Implementation Decree
Modalities of payment	Monthly payment, guaranteeing (family of) beneficiary an income at the level of social assistance in NL, adapted to cost of living in country of origin	As before, with the addition of a health insurance allowance	As before	Yearly payment, calculated as a function of the beneficiary’s income	Yearly payment, topping up beneficiary’s annual income to €6600
Nationality conditions	TCNs belonging to groups covered by the government’s minorities policy	As before, but EU citizens from former recruitment states are also eligible	As before, but EU citizens from former recruitment states are eligible only if they arrived in NL before TEU came into force in their country of origin	All TCNs; EU citizens are ineligible	All foreign nationals, including EU citizens
Age conditions	Aged at least 55 years	Aged at least 45 years	Aged at least 55 years	Aged at least 65 years, or 60 years if unfit to work	As before
Continuous prior residence conditions	At least 5 years’ continuous prior residence in NL	At least 3 years’ continuous prior residence in NL	At least 8 years’ continuous prior residence in NL	At least 15 years’ continuous prior residence in FR; must be resident in a migrant worker hostel at the time of application	As before, but 15 years’ condition does not apply to EU citizens
Other conditions	Claiming unemployment or disability benefit for at least 6 months	As before	Claiming unemployment or disability benefit for at least 12 months	None	Annual income must be less than €6,600; the beneficiary must be in receipt of all other pension income which he is entitled to claim
Type of return	Permanent return; the aid is revoked if the beneficiary returns to live in NL	As before	As before	Semi-permanent return; the aid is for those who wish to undertake extended visits in their country of origin	As before, with the specific requirement that beneficiaries must spend more than 6 months per year in country of origin

## The Dutch remigration scheme^4^

The Dutch scheme started as a pilot scheme in 1985 and has since been adapted several times. As Table 1 shows, eligibility criteria and requirements for the scheme were relaxed during the first half of its lifetime but have since been tightened. Although its manifest aim has remained unchanged—to make return migration possible for older migrants with poor prospects in the Netherlands—a tacit budgetary purpose of the scheme has gained in importance, and the departure from the logics of the welfare state has come to encounter more opposition during the past 15 years, as the political climate towards immigrants grew colder.

The remigration scheme was introduced at a time when many migrants who had been recruited as ‘guest workers’ in the 1960s and 1970s lost their jobs and there was little prospect of re-integrating them in the Dutch labour market. The government—a coalition of Christian-Democrats and liberal-conservatives—had just adopted a ‘minorities policy’ to promote the socio-economic integration of these labour migrants (and their spouses and children) and other immigrant minorities (immigrants from former colonies and refugees). The remigration scheme was seen as the final element of this policy, offering older unemployed migrants (aged 55 and over) who stayed in the Netherlands only because they had no means of living in their home country, the possibility to return home with a monthly benefit. Although the scheme was meant primarily for ‘guest workers’, other immigrant minorities were also eligible. Migrants with Dutch citizenship, however, were excluded because they could not be prevented from returning to the Netherlands. For the same reason, migrants from EC Member States were also excluded. According to the government, it was reasonable to make the offer of lifelong income security conditional on the migrant’s leaving the Netherlands for good. This condition could not be enforced on Dutch and EC citizens.

Immigrant organisations and parliament had pressed the government to remove obstacles to exporting unemployment benefits and assistance from the existing social security legislation. Some had pressed for a scheme similar to the one that was in force in Germany in 1983–1984. This would entail that returning migrants got the social security contributions they had paid in the Netherlands refunded.^5^ However, an advisory body to the minister responsible for social security matters advised against fundamental changes to the existing social security system and the government followed this body’s advice to put in place a new scheme targeted specifically at migrants who had poor prospects in the Netherlands and who desired to return to their country of origin. Although the decision to introduce such a scheme was based on what could be called ‘paternalistic pragmatism’, the limitation of the target group to recipients of unemployment or disability benefits and the requirement that the return be permanent were clearly to the benefit of the Dutch welfare budget. This budget-saving rationale was not mentioned explicitly though.

The pilot remigration scheme became permanent in 1987 and in 1999 was given a statutory basis with the adoption of the Remigration Act. The bill for the Remigration Act proposed several improvements for potential users and parliament amended the bill to introduce several other improvements and to extend the target group to include EU citizens from former recruitment states. The latter amendment was adopted after extensive debate over its compatibility with EU law. The government thought it ‘legally vulnerable’ to distinguish between citizens of former recruitment states and other EU citizens. A majority in parliament insisted that former guest workers from Italy, Spain, Portugal and Greece should be treated equally to former guest workers from Turkey and Morocco. Finally, it was decided that including the Southern Europeans would not constitute an unlawful distinction, because these groups had been designated as target groups of the government’s minorities policy on the basis of an ‘objective, non-discriminatory criterion’ (namely their disadvantaged socio-economic position).

Three years after parliament had passed the Remigration Act, a bill was introduced to repeal it. According to the new, conservative-liberal minister for Aliens’ Affairs and Integration Rita Verdonk, return migration was the responsibility of the migrant, not that of the government, and the termination of the remigration scheme would save the government tens of millions of Euros. The latter argument turned out to be invalid, however. A study commissioned by immigrant organisations showed that over a period of 10 years, the government and the social security funds would save nearly 400 million Euros by keeping the remigration scheme in place (Berkhout 2003). Parliament subsequently passed a motion requesting the government to withdraw the repeal bill. However, it was not long until the remigration scheme became debated again. On the one side, the European Commission had doubts about its compatibility with the free movement of persons (as it was not possible to return to the Netherlands while retaining the remigration benefit) and the principle of equal treatment (as the scheme could not be used by all EU citizens). On the other side, a majority in parliament considered the scheme to be outdated. Due to several changes of government, it was not until the end of 2011 that a bill to revise the Remigration Act was introduced. The explanatory memorandum stated that the proposed revisions were not motivated by economic considerations but by the desire to promote the integration of immigrants and to retain older migrants in the work force. Along with several tightenings, the bill proposed to let the scheme expire in 2024. It proposed to meet the objections of the European Commission by providing that migrants from former recruitment countries in Southern Europe would be eligible only if they had arrived in the Netherlands before the entry into force of the Treaty on European Union in their country of origin. The bill passed both chambers of parliament in 2013. The revised Remigration Act entered into force on 1 July 2014.

## The French aid for familial and social reinsertion scheme

In 2004, Prime Minister Jean-Pierre Raffarin requested a written opinion on the social situation of older migrant workers in France from the High Council for Integration (HCI), a government agency set up to monitor migrant integration issues.^6^ The HCI’s subsequent report highlighted the constraints on older migrants stemming from eligibility criteria for non-contributory welfare benefits, such as the 6-month residence period required to receive old-age income support. In this regard, the HCI ruled that the residence requirements constituted a ‘de facto inequality’ insofar as foreigners were over-represented among the recipients of old-age income support, leading some to not return home although they wished to do so (HCI 2006: p. 126). Furthermore, the report noted that continuing to host this elderly and increasingly infirm population imposed a high financial cost on the French state. Particularly alarming for the HCI was the situation of ‘geographically single’ male migrants in migrant worker hostels (*foyers de travailleurs migrants*), living far from their wives and children whom they continued to support financially in countries of origin in North and West Africa.^7^ Older hostel residents are in this respect exemplars of transnational ageing, implicated economically, socially and sometimes politically both in France and places of origin, maintaining two households, speaking two (or more) languages, and incessantly travelling back-and-forth across the Mediterranean (Hunter 2016).

The vulnerable situation of older hostel residents was not only a concern of government during the mid-2000s. There was growing public consciousness of the iniquities which older and particularly North African migrants (*chibanis*) living in hostels faced, as shown by print and broadcast media depictions of hostels as sub-standard housing entirely unsuitable for older people. *Indigènes*, a commercially successfully and critically acclaimed 2007 French-language film about the discrimination suffered by North African soldiers who fought for France in WW2, contributed to this narrative in its portrayal of the hostel where one of the now-elderly protagonists lives. The film’s release prompted the government to partially redress discriminatory treatment of foreign veterans, namely to unfreeze their military pensions which had been paid at a lower rate than French veterans since 1959.

It was in this broader climate of what can be called ‘institutional repentance’ that the Aid for Familial and Social Reinsertion was born, driven by Jean-Louis Borloo, Minister of Employment, Social Cohesion and Housing from 2004 to 2007. The stated motivations of the creators of the Aid were two-fold.^8^ Firstly to give hostel residents more freedom as regards where to spend their retirement, by no longer requiring them to spend at least 6 months per year in France in order to receive old-age income support; and secondly to recognise the ‘sacrifices made by these workers for the economic development of France’ (République Française 2007). Rachid Bouzidi, special adviser to Borloo, explained in a newspaper interview what had prompted them to draft the bill:

Quite simply the fact that in the cabinet of Jean-Louis Borloo we believe that everyone has the right to live with his family in a decent and dignified way. We believe that it is abnormal that *chibanis* remain in France against their will, only to retain their modest incomes and access to health. These men primarily arrived in the 1960s to work, because France asked them to. At that time, not a road, not a bridge, not a building was built without their help. So today, to permit them to live as they wish is not special treatment. It’s simply the right thing to do. (Raouf 2007)

In addition, although not expressed in publicly available documents at the time, Borloo has since acknowledged a budgetary rationale for the Aid, as hinted also by the HCI report. In his testimony to the Parliamentary Mission on Older Migrants in 2013, he described the Aid as ‘a gesture of Republican dignity which moreover would cost France nothing… One might even regard [the *chibani’s*] return visits home as generating savings for our public services, notably in health’ (Bachelay 2013).

The drafting of the bill took over a year and was introduced to parliament in January 2007. The text was debated in both houses of the legislature. One of the sticking points in parliament concerned the reversibility of the Aid in case claimants no longer wished to undertake extended return visits to their country of origin. An amendment was passed to enable beneficiaries to resume their former entitlements to housing and income support in case of renunciation of the Aid. A further amendment was introduced in parliament after concerns were raised that ARFS beneficiaries visiting France would be deprived of access to the subsidised healthcare which they had previously benefited from. Indeed, maintaining continuity of care and relationships with trusted doctors is a major factor behind older hostel residents’ transnational lifestyles (Hunter 2016). A clause was added to Article L.311-7 of the Social Security Code guaranteeing access to French health services in this event. The text was passed by unanimous vote—a rare event—in both the *Assemblée Nationale* and *Sénat* in February 2007 and entered the statute book under Article L. 117-3 of the Social Action and Families Code. The main eligibility criteria for the Aid as per the 2007 parliamentary act are summarised in Table 1.

Although voted by parliament, the act could not enter into force until an Implementation Decree had been drafted by the ministries concerned and approved by the *Conseil d’Etat*, a legal oversight body. This proved much more difficult than expected, as the 8-year hiatus between parliamentary vote (2007) and implementation decree (2015) indicates. There were two principal sources of delay. The first concerns the unexpected legal complexity of implementing the Aid, particularly at the level of European law. The law passed by parliament in 2007 risked contravening non-discrimination clauses in the EU treaties since it only concerned third-country nationals and not EU nationals, some of whom do reside in hostels. A second complication arose from Regulation 883/2004, which stipulates that previous periods of insurance, work or residence in other member states will be taken into account when calculating an individual’s benefits. As noted by the special rapporteur for the 2011 Immigration, Asylum and Integration budget (Commission des finances 2011), in the case of the ARFS the extension of residence to all member states ‘poses problems both as regards the effectivity of this residence condition in one of the member states (in practice impossible to verify) and as regards the cost of the scheme’. The worry was that in rendering the ARFS compatible with Regulation 883/2004 the Aid would become accessible to a much wider public, leading to ballooning costs: put simply, any third-country or EU national aged over 65 having lived in any EU member state for 15 years or more would be eligible for this return aid if they moved to live in a hostel in France for a short period of time.

Given these risks, there was unsurprisingly ‘a lot of reluctance to secure a decree [for this law]’, according to a union leader in this sector. This brings us to the second source of delay in implementing the Aid, namely the vagaries of electoral politics. Shortly after the passage of the bill, France experienced a change of President and government. The incoming centre-right government was keen to promote a harder line on immigration in general, and was therefore less favourable to the law, according to Alexis Bachelay MP, rapporteur of the 2013 Parliamentary Mission on Older Migrants. The new Minister for Immigration, Integration, National Identity and International Development, Brice Hortefeux (2007-9), was ‘charged with burying’ the Aid (ATMF 2014: pp. 24–25), an allegation reinforced by testimony from Borloo to the 2013 Parliamentary Mission (Bachelay 2013). Yannick Imbert, director of the government agency responsible for the reception and integration of new migrants and asylum seekers (OFII), blamed the government’s stance on electoral considerations: ‘the possibility to benefit from the aid while at the same time settling definitively in the country of origin could have posed … a political problem: would the French public accept that people no longer residing in our country could continue to receive benefits from French agencies?’ (Bachelay 2013: p. 201).

As a result of these two obstacles—electoral considerations and EU law—the legislation was effectively shelved after 2007. It was only following the victory of the Socialist Party (PS) in the 2012 presidential and legislative elections that the Aid was re-established on the political agenda. This was in large part thanks to the Parliamentary Mission on Older Migrants in 2013, created at the behest of the incoming President of the Assemblée Nationale, Claude Bartolone (PS). One of the Mission’s key recommendations—discussed at length in the Executive Summary—called on the government to publish the implementation decree for the ARFS. Nonetheless, a further 2 years passed before an acceptable solution, compatible with EU law, was found, indicating that even with political support the legal complexities were significant. As is summarised in Table 1, the main difference between the 2015 Implementation Decree and the text of the 2007 Act is that the Aid is now open to all foreign nationals, whereas previously EU citizens were excluded. This renders the Aid compatible with non-discrimination clauses in the EU treaties. Given the concerns arising from the principle of ‘aggregation of periods’ in Regulation 883/2004, EU nationals are not subject to the requirement of 15 years’ continuous prior residence in France. Lastly, the 2015 Decree requires beneficiaries of the Aid to be in receipt of all other pension income to which they are entitled, both in France and elsewhere, and that only those whose total annual income is lower than €6600 may apply for the Aid. It seems very likely that these latter measures were designed to minimise the attractiveness of the Aid to individuals currently living outside France.

## Discussion

Transnational ageing has self-evident implications for social protection, and in this paper we have analysed two policies which offer financial aid to older migrants who might otherwise not be able to afford relocation to their place of origin. These two schemes differ from other return aids which thus far have received scholarly attention, in that what is on offer is not a one-off lump sum but rather a guaranteed lifelong income. The two schemes thus bear a strong family resemblance to non-contributory welfare benefits such as old-age income support, and we have analysed them according to the logics of the welfare state, namely solidarity, territoriality and nationality.

Both migration scholars and social security scholars have argued that welfare states are inherently closed systems: the principle of solidarity can only be upheld if benefits are preserved for nationals or permanent residents who reside or work on the state territory (De Swaan 1995; Freeman 1986; Sciortino and Finotelli 2015; Van der Mei 2003; Vonk 2002). The French and Dutch schemes, however, are open only to non-nationals and only to those non-nationals who no longer reside permanently on national territory. We set out to address the puzzling question of why policymakers initiated legislation which undermines the welfare state principles of territoriality and nationality, particularly at a time when in other respects we are witnessing welfare retrenchment. Are the prospects for transnational social protection more favourable than proponents of the closed-system thesis have predicted?

Both the French and the Dutch scheme were put in place for specific groups of older migrants, to whom the state felt a special responsibility. In France, the ARFS was a manifestation initially of what we termed ‘institutional repentance’, namely an acknowledgement of migrant workers’ contributions to France’s economic development and their poor living conditions in state-sanctioned hostel accommodation. The discourse about the ‘sacrifices’ of the ageing hostel residents suggests that legislators were motivated in part by symbolic considerations. The Dutch scheme had similarly benign origins, which we labelled ‘paternalistic pragmatism’: to facilitate return for former ‘guest workers’ who had lost their jobs and whose prospects in the Dutch labour market were poor. However, both the French and the Dutch governments sought to leave the existing social protection system intact and to limit the target group of the special scheme. Changes in government and the political saliency of immigration were also a feature in the development of both schemes. The French ARFS was shelved for 8 years. During this time, the issue of the territoriality of the welfare state was raised as a motive for opposition to the scheme. The Dutch scheme was all but repealed after a change of government; a budgetary rationale became decisive for its retention. A few years later, however, a majority in parliament considered the scheme to be outdated and agreed to let it expire. The budgetary savings were no longer an argument to retain the scheme, as the dominant political idea now was that older workers, immigrant workers included, should be retained in the work force. Another feature in the development of both schemes was that the need to render the schemes compatible with EU law was a major preoccupation of the policymakers—a paradoxical feature insofar as the EU free movement and equal treatment rules are meant to facilitate mobility. However, it becomes less paradoxical when one realises that the compatibility difficulties were in large part due to the Dutch and French policymakers’ wish to keep the schemes highly targeted and to prevent fundamental opening of the national welfare system. In sum, in both cases the departure from the logics of the welfare state was not fundamental.

Given the prevalence of transnational ageing and the recent increased academic attention to it, now seems an opportune moment to ask what the prospects are for similar policies in other countries where significant numbers of older migrants live. Given the legal complexities, particularly in an EU context, and the potential for political opposition based on unease at undermining welfare state principles of territoriality and nationality, one might expect there to be little appetite among policymakers to follow the trail blazed by the Netherlands and France in this regard. Nonetheless, such policies can be a win–win, both for governments and for migrants. For governments, political opposition may be neutralised by the budgetary savings generated by these schemes, since older returnees do not consume public services such as healthcare. This argument has proven very effective in the Netherlands as our case study showed. Migrants may gain from such schemes insofar as a wider set of options for returning on a permanent or semi-permanent basis becomes financially envisagable. Nevertheless, it bears repeating that residence choices at retirement are not only decided according to an economic cost-benefit analysis. Rather the decision to age transnationally must also be reconciled with additional and sometimes competing priorities: access to healthcare, the location of different family members, care responsibilities to younger or older relatives, cultural and/or religious norms, as well as more existential questions of identity and belonging.

## Footnotes

1 We use the term social protection to refer to both social assistance (or welfare) and social insurance mechanisms.

2 Own calculations based on data from Statistics Netherlands (http://statline.cbs.nl/Statweb/).

3 These households were receiving a Dutch (disability or old-age) pension which exceeded the remigration benefit. They would receive a remigration benefit if this pension was stopped or reduced.

4 This section is based on an analysis of parliamentary documents and debates. The following dossiers were studied: 18939 (Remigratie); 29020 (Intrekking van de Remigratiewet); 33085 (Wijziging Remigratiewet).

5 For more information on the German return incentive programme, see Martin 1991, pp. 39–40.

6 This letter is reproduced in HCI 2006: pp. 47–48.

7 For more information on the migrant worker hostels and their ageing clientele, see Hunter (2015).

8 More sceptical observers have put forward a third rationale: to encourage older migrants to leave the hostels, thereby liberating this accommodation for vulnerable priority publics such as homeless people (Dimier 2007).
